# Alpha-adrenergic antagonists and iris dynamics: Challenges and solutions in cataract surgery

**DOI:** 10.1186/s12886-024-03705-1

**Published:** 2024-10-03

**Authors:** Jaya Kaushik, Rishi Sharma, Sumit Goyal, Meenu Dangi, Rakesh Kumar Jha, Ankita Singh

**Affiliations:** 1 Dept of Ophthalmology, Command Hospital (CC), Lucknow, India; 2 Department of Ophthalmology, Military Hospital, Bareilly, India; 3 Dept of Ophthalmology, Military Hospital, Wellington, Ooty India; 4 Department of Ophthalmology, Military Hospital, Bathinda, India

**Keywords:** Alpha adrenergic antagonists, Tamsulosin, Pupillary diameter, Intraoperative floppy iris syndrome (IFIS)

## Abstract

**Background:**

Alpha-1 adrenergic receptor antagonists (α1-ARAs) are frequently used in treatment of Hypertension and symptomatic benign prostatic hypertrophy (BPH). Numerous studies have demonstrated the association between α1-ARAs like Tamsulosin and increased surgical risks for patients undergoing cataract surgery. This study aims to identify and study the effects of α1-ARAs on iris parameters and the subsequent operative challenges encountered during cataract surgery.

**Methods:**

A cross-sectional, prospective study involving 30 patients on α1-ARAs planned for cataract surgery and equal number of age and sex matched controls were subjected to evaluation of changes on iris parameters and subsequent challenges in cataract surgery.

**Results:**

The study group had statistically significant lesser pupil diameter. Iris thickness at sphincter muscle region (SMR) was similar between groups (*P* = 0.53). Significantly lower values of iris thickness at dilator muscle region (DMR) found in treated subjects (P = < 0.001). There was statistically significant difference between DMR/SMR ratio of two groups (*P* < 0.001). Multiple regression analysis revealed longer duration of α1-ARAs treatment correlated with reduced DMR/SMR ratio (*P* = 0.001; *r* = 0.47).

**Conclusion:**

α1-ARAs have implications for pupil size regulation and surgical procedures involving the eye. Tamsulosin is more potent than alfuzosin in inducing IFIS. Systemic α1-ARAs lower values of DMR thickness, DMR/SMR ratio and reduces pupillary diameter. Therefore, ophthalmologists, primary care physicians, urologists, and patients should be aware of the potential difficulties that these drugs pose for cataract surgery.

## Introduction

Alpha-1 adrenergic receptor antagonists (α1-ARAs) or Alpha-1 blockers consist of drugs that block the effect of catecholamines on alpha-1 adrenergic receptors in smooth muscle tissues. They are commonly used in the management of benign prostatic hyperplasia (BPH), hypertension and post-traumatic stress disorder [1]. BPH involves the use of tamsulosin which is a selective α_1A_-ARA that helps in alleviating urinary symptoms.

However, their pharmacological action extends to ocular tissues, more precisely, the smooth muscle of iris dilator muscle, leading to side effects that are particularly significant during cataract surgery making it more challenging with increased risk of complications [2]. The mechanism by which α1-ARAs induce these changes involves the inhibition of alpha-1 receptors in the iris dilator muscle, resulting in reduced muscle tone and, consequently, smaller pupil diameters with decreased iris rigidity. The implications of these effects in ophthalmology are relevant with the increasing prevalence of primary users of these medications among the aging population who are undergoing cataract surgery.

One of the most characteristic ocular side effects associated with α1-ARAs is intraoperative floppy iris syndrome (IFIS), first described by Chang and Campbell in 2005 [3]. It is characterized by a triad of symptoms: fluttering and bellowing of iris stroma, propensity for the pupil to constrict during surgery, and tendency for the iris to prolapse toward the incisions. This syndrome complicates phacoemulsification by making it more challenging for the surgeon to operate, thus increasing the risk of intraoperative and postoperative complications.

Schreiner et al. (2009) documented that patients on tamsulosin present with significantly smaller preoperative pupil sizes, necessitating additional surgical interventions [4]. Blouin et al. (2007) noted that while alfuzosin and other α1-ARAs are less frequently associated with IFIS than tamsulosin, they still pose a considerable risk for intraoperative complications [5]. Numerous other studies have demonstrated the association between selective α1-ARAs drugs like Tamsulosin and increased surgical risks for patients undergoing cataract surgery [3, 6].

Understanding the impact of α1-ARAs on iris parameters is crucial for planning and executing cataract surgery. Awareness and appropriate management strategies can mitigate risks and improve surgical outcomes. In this study we aim to identify and study the effects of α1-ARAs on iris parameters and the subsequent operative challenges encountered during cataract surgery as a result of morphological changes induced in the iris by these agents. By providing a comprehensive analysis of these effects and discussing effective management strategies, this research seeks to enhance the surgical approach and outcomes for patients on these drugs.

### Aim and objectives

Study the effects of α1-ARAs on iris parameters and subsequent challenges in cataract surgery.

## Materials and methods

### Study design

This prospective review was conducted for 60 patients, taken from general ophthalmology outpatients department, who were planned cataract surgery. This evaluation was conducted after dividing the study participants into two groups. The first / study group included thirty patients on α1-ARAs and thirty similar age-sex matched controls were inducted in the second / control group. The participants were randomly enrolled into 2 groups using random number tables.

*Inclusion criteria were*:

1. Diagnosis of cataract requiring surgical intervention.
2. Use of α1-ARAs (e.g., tamsulosin, alfuzosin) for at least three months prior to surgery.
3. Availability of complete medical records and follow-up data.

*Exclusion criteria included*:

1. Previous intraocular surgery.
2. Co-existing ocular pathologies (e.g., glaucoma, uveitis).
3. Use of other medications affecting pupil size (e.g., anticholinergics).

*Strategies to remove confounding*:

1. All cases performed by same surgeon (right handed).
2. All surgeries carried out using topical anaesthesia.
3. 2.8 mm main entry port and 15-degree side port was used in all cases.

### Data collection

Anterior segment was examined by means of slit lamp. Detailed mydriatic fundus examination was done by 90D slit lamp bio-microscopy, direct and indirect ophthalmoscopy.

Data collected included demographic information (age, sex), duration and type of alpha-adrenergic antagonist use and iris characteristics, intraoperative complications (e.g., IFIS, iris prolapse, zonular dehiscence), surgical duration, postoperative outcomes (e.g., BCVA, intraocular pressure, complications). Sirius Scheimpflug tomographer was used to study photopic pupillary diameter preoperatively.

**Fig. 1:**
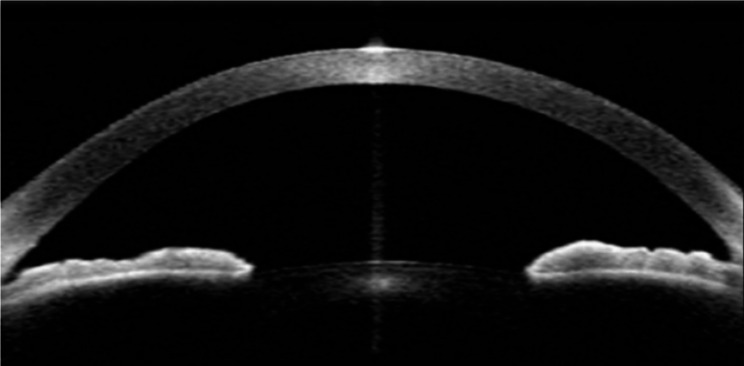
AS-OCT image of anterior chamber angle of a patient from study group

**Fig. 2:**
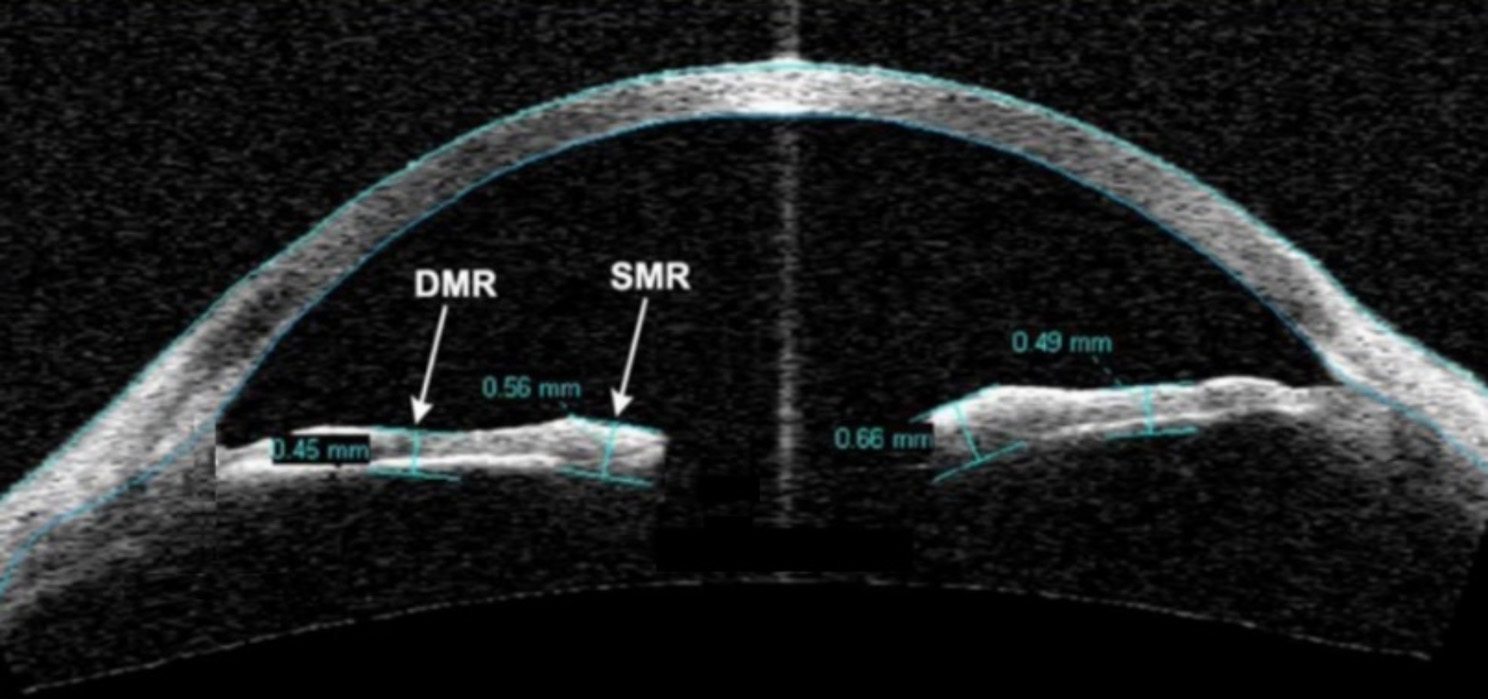
Showing measurement of SMR (0.75 mm from the pupillary margin) and DMR (measured at half distance between the scleral spur & the pupillary margin) on AS-OCT

Using anterior segment optical coherence tomography (AS-OCT), dilator muscle region (DMR) iris thickness measured at half distance between the scleral spur & the pupillary margin and sphincter muscle region (SMR) iris thickness measured 0.75 mm from the pupillary margin (Figs. 1 and 2). The ratio between the DMR/SMR to compensate for possible intersubject variability and pupillary diameter were also studied.

### Statistical analysis

The recorded data was analysed using commercial software (SPSS Version 24.0; SPSS, Inc., Chicago, IL, USA). Descriptive statistics to summarize clinical characteristics and patient demographics. The *t*-test was used to compare the pupillary diameter and DMR/SMR ratio in patients on α1-ARAs and control group. The pupillary diameter and DMR/SMR ratio width was correlated with the duration of treatment with α1-ARAs. A *p*-value of < 0.05 were considered statistically significant.

#### Ethical issues

This study adheres strictly to the tenets of the Declaration of Helsinki. All aspects of this study were vetted by the institutional ethical committee. All patients signed an informed consent.

Title of the committee- Institutional Ethics Committee.

Affiliation- Command Hospital (Central Command), Lucknow − 226,002 (UP).

Country- India.

Approval number- IEC Registration No: EC/NEW/INST/2021/2471.

## Results

The mean age of the study group was 70.9 ± 7.5 years (*n* = 30). The mean age of control group was 67.1 ± 8.1 years (*n* = 30). There was no statistically significant difference between the two groups (*P* = 0. 061).

Both the groups had 18 (60%) males and 12 (40%) females. Gender distribution was statistically similar and insignificant in both the groups (*p* = 1.000). Eye color and laterality were not significant.

### Drugs usage

In the study group, out of 30 participants, 27 patients were on Tamsulosin, two patients were on Alphazosin and one patient was on Tamsulosin and Alphazosin combination therapy.

**Fig. 3:**
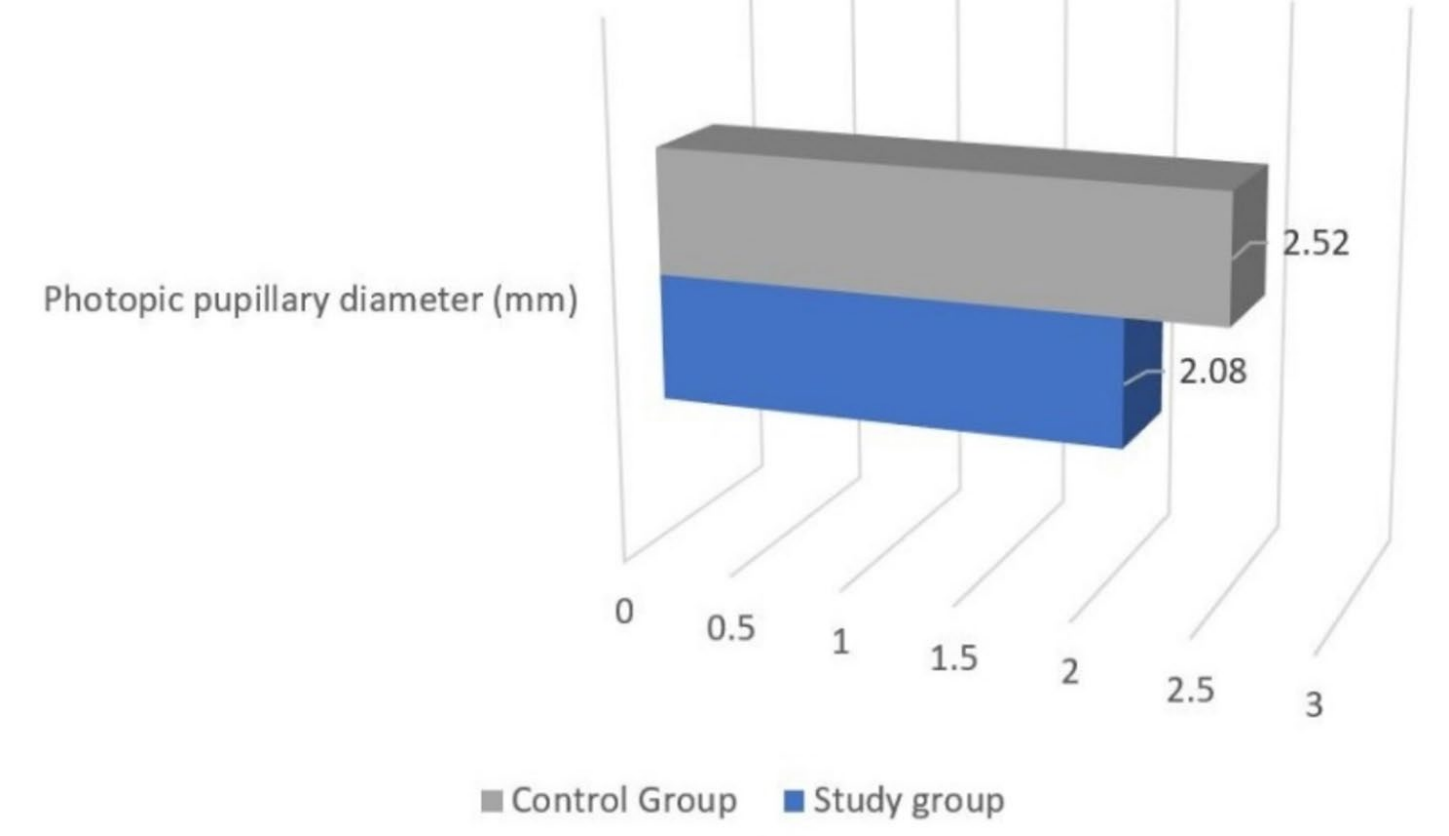
Comparison of photopic pupillary diameter between the two groups

Photopic pupillary diameter was reduced in the study group 2.08 ± 0.8 mm when compared to the controls wherein it was 2.5 ± 0.6 mm. (*p* = 0.0001) (Table 1; Fig. 3).

### DMR, SMR and DMR/SMR ratio

**Fig. 4:**
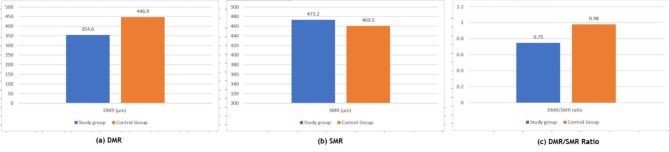
Comparison of DMR, SMR and DMR/SMR ratio between both groups

Significantly lower values of DMR found in treated subjects whose mean was 354.6 ± 83.7 μm as compared to mean of 446.9 ± 92.6 μm in controls (P = < 0.001) (Fig. 4). There was no statistically significant difference between means of SMR for study group (473.2 ± 76.5 μm) and control group (460.5 ± 99.5 μm) (*P* = 0.530). There was statistically significant difference between DMR/SMR ratio of the two groups (*P* < 0.001) (Table 1).

**Table 1:** Comparison of demographic and morphological data between both groups

Parameter	Study group, *n* = 30 (Range)	Control Group. *n* = 30 (Range)	*P*-value
Age	70.9 ± 7.5	67.1 ± 8.1	0.061
Photopic pupillary diameter (mm)	2.08 ± 0.8 (1.28–3.2)	2.52 ± 0.6 (1.7–3.9)	0.0001
**Iris thickness measurements**			
DMR (µm)	354.6 ± 83.7 (176.7–522)	446.9 ± 92.6 (291.3–611)	< 0.001
SMR (µm)	473.2 ± 76.5 (359.3 681.3)	460.5 ± 99.5 (328.3–745)	0.530
DMR/SMR ratio	0.75 ± 0.2 (0.36–1.07)	0.98 ± 0.1 (0.79–1.23)	< 0.001

SMR: Sphincter Muscle Region; DMR: Dilator Muscle Region

### Duration of treatment for Tamsulosin

Out of 27 patients, 20 were on Tamsulosin for last 10 years, five patients since 05 years and two patients since 03 years. Multiple regression analysis revealed that a longer duration of α_1A_-ARA treatment correlated to a reduced DMR/SMR ratio (*P* = 0.001; *r* = 0.47).

### Events during cataract surgery

Out of 30 participants in study group, 28 patients were taken up for cataract surgery. Intraoperatively, 22 patients (78.57%) had IFIS, two patients (7.14%) developed posterior capsular rent (PCR) and four patients (14.28%) had iris chaffing. Two patients were excluded from the study as one patient developed conjunctivitis and the other one had raised blood sugar levels just before surgery. Hence, their surgery was cancelled. In the control group, all 30 patients underwent cataract surgery with no intraoperative IFIS, PCR or iris chaffing.

## Discussion

α1-ARAs are frequently used in treatment of Hypertension and symptomatic Benign prostatic hypertrophy (BPH) [7]. Of the three subtypes of alpha-1 receptors (a, b, and d), the alpha-1a receptor dominates in the iris dilator muscle as well as in the smooth muscles of the prostate tissue. Also, the beta Tamsulosin is subtype specific and has affinity 20 times for the alpha-1a receptor [8]. The pupillary dilatation is severely impeded by the use of α1-ARAs, however, the frequency and severity of pathological alterations in the iris morphology is most marked with use of Tamsulosin (α_1A_-ARA) [9–11]. Photopic pupillary diameter was studied for all comparisons as studying the impact of using α1-ARAs on pupillary diameter especially under photopic conditions encountered during cataract surgery is of utmost importance. Our observations regarding the reduction in photopic pupil diameter and iris rigidity among patients receiving α1-ARAs are consistent with existing literature.

Tzamalis A et al. in their study concluded that the rate of intraoperative events in known cases of IFIS is higher amongst females as compared to males, however, the overall incidence of IFIS is significantly higher in males [12]. There is scientific evidence establishing the association between the use of α1-ARAs in IFIS [13], a term, coined by Chang and Campbell in 2005 [1]. These are the possible alterations resulting in the clinical entity of IFIS. This consists of fluttering and bellowing of iris stroma, propensity for the pupil to constrict during surgery, and tendency for the iris to prolapse toward the incisions. Few studies have mentioned that IFIS has been noted even in patients who had stopped tamsulosin 2 years before cataract surgery [3, 14]. However, minimum duration of intake of tamsulosin leading to IFIS has not been concretely established by any of the studies.

In our study the results show marked changes in the iris morphology in patients with a past or current history of α1-ARAs usage as compared to age-matched controls. There was statistically significant decrease in the DMR thickness, lower DMR/SMR ratios and smaller pupillary diameters. This is in consonance with the first report by Prata TS et al. [10] which demonstrated structural alteration in the iris dilator muscle region in patients using α1-ARAs. We found no significant difference in the SMR thickness which showed no statistically significant difference between the study patients and control group. Altered iris morphology was present in 68% of treated patients compared to controls. This figure is in sync with the reported prevalence of clinical IFIS in 62.5–93.8% patients on tamsulosin or with history of tamsulosin use [15–18]. A study by Christou CD et al. revealed strong correlation of Silodosin with IFIS development and suggested the use of appropriate prophylactic measures to its impact on the surgical outcome [19].

These alterations in iris can lead to myriad of complications that range from poor visibility of the operative field, iris damage, posterior capsule rupture and posterior dislocation of lens material intraoperatively. The surgical challenges posed by IFIS and other complications related to α1-ARAs necessitate specific management strategies.

Strategies such as medication review, preoperative discontinuation of alpha-blockers when feasible and modification of surgical techniques can help optimize outcomes and minimize complications. Preoperative planning is crucial, including a thorough medication review and consideration of discontinuing α1-ARAs prior to surgery when feasible. However, this approach must be balanced against the potential risks of interrupting treatment for the underlying condition. Alternative medications, such as non-selective α-ARA, may also be considered in consultation with the patient’s primary care provider. A study by Chang DF (2008) suggested preoperative administration of alpha-adrenergic agonists may help improve iris tone and mitigate the risk of IFIS during surgery [20]. Similar study by Bucci Jr FA, et al. (2012) found that preoperative use of topical phenylephrine and ketorolac combination was effective in reducing the severity of IFIS and improving surgical outcomes in patients on tamsulosin [21].

Intraoperative management techniques are equally important in preventing or addressing the potential complications. The use of mechanical devices such as iris hooks or rings, like the Malyugin ring, can help maintain pupil dilation and stability during surgery. Viscomydriasis, which involves the injection of a cohesive viscoelastic agent, can also aid in maintaining pupil size and preventing iris prolapse. Additionally, modifications in phacoemulsification techniques, such as adjusting fluidics settings and carefully manipulating the iris, can help manage its instability and reduce the risk of complications.

While this study provides valuable insights into the effects of α1-ARAs on iris parameters and cataract surgery outcomes, several limitations must be acknowledged. The nature of the study design introduces the potential for selection bias and limits causal inference. Future prospective studies with larger sample sizes are needed to validate our findings and elucidate the underlying mechanisms driving medication-specific differences in IFIS risk. Furthermore, our study did not assess the impact of α1-ARAs on postoperative complications or long-term visual outcomes as it primarily focused on the immediate perioperative period. To evaluate their implications on long-term ocular health and visual function, longitudinal studies with extended follow-up periods are warranted. Additionally, studies evaluating comparative effectiveness of different management strategies for IFIS, including preoperative medication review, intraoperative techniques, and postoperative care protocols, are needed to establish evidence-based guidelines to further optimize surgical outcomes.

## Conclusion

In conclusion, appropriate pre-operative assessment which includes history of BPH or HTN treatment especially in elderly patients should be elicited as α1-ARAs have a significant impact on iris behavior, leading to increased challenges in cataract surgery, particularly due to IFIS. Systemic α1-ARAs lower values of DMR thickness, DMR/SMR ratio and reduces pupillary diameter. Severity of pathological alterations in iris morphology is most marked with use of Tamsulosin (α_1A_-ARA). Communication among the treating physicians about treatment with α1-ARAs is essential and discontinuation of α1-ARAs at least 1 week prior to cataract surgery may be considered.

## Data Availability

The datasets generated and/or analysed during the current study are not publicly available due to institutional protocols and norms, but are available from the corresponding author on reasonable request.

## Abbreviations

α1: ARAs-Alpha-1 adrenergic receptor antagonists
BPH: Benign prostatic hypertrophy
SMR: Sphincter muscle region
DMR: Dilator muscle region
IFIS: Intraoperative floppy iris syndrome
AS: OCT-Anterior segment optical coherence tomography
BCVA: Best corrected visual acuity
PCR: Posterior capsular rent

## References

[1] Nickel JC, Méndez-Probst CE, Whelan TF, Paterson RF, Razvi H. 2010 update: guidelines for the management of Benign Prostatic Hyperplasia. Can Urol Association J. 2013;4:310–16. 10.5489/cuaj.10124 PMC2950766 20944799

[2] Chatziralli IP, Sergentanis TN. Risk factors for intraoperative floppy iris syndrome: a meta-analysis. Ophthalmology. 2011;118(4):730–5. 21168223 10.1016/j.ophtha.2010.08.039

[3] Chang DF, Campbell JR. Intraoperative floppy iris syndrome associated with tamsulosin. J Cataract Refract Surg. 2005;31(4):664–73. 15899440 10.1016/j.jcrs.2005.02.027

[4] Schreiner B, Hengerer F, Dick HB. Impact of tamsulosin on pupil diameter and intraoperative complications during cataract surgery. Am J Ophthalmol. 2009;148(4):504–10.

[5] Blouin MC, Blouin J, Perreault S, Lapointe A, Dragomir A. Intraoperative floppy-iris syndrome associated with alpha1-adrenoreceptors: comparison of tamsulosin and alfuzosin. J Cataract Refract Surg. 2007;33(7):1227–34. 17586379 10.1016/j.jcrs.2007.03.032

[6] Chang DF, Campbell JR, Colin J, Schweitzer C, Study Surgeon Group. Prospective masked comparison of intraoperative floppy iris syndrome severity with tamsulosin versus alfuzosin. Ophthalmology. 2014;121(4):829–34. 24314842 10.1016/j.ophtha.2013.10.031

[7] Foglar R, Shibata K, Horie K, Hirasawa A, Tsujimoto G. Use of recombinant alpha 1-adrenoceptors to characterize subtype selectivity of drugs for the treatment of prostatic hypertrophy. Eur J Pharmacol. 1995;288(2):201–7. 7536677 10.1016/0922-4106(95)90195-7

[8] Hieble JP, Bylund DB, Clarke DE, et al. International Union of Pharmacology. X. recommendation for nomenclature of alpha 1-adrenoceptors: consensus update. Pharmacol Rev. 1995;47(2):267–70. 7568329

[9] Pärssinen O, Leppänen E, Keski-Rahkonen P, Mauriala T, Dugué B, Lehtonen M. Influence of tamsulosin on the iris and its implications for cataract surgery. Invest Ophthalmol Vis Sci. 2006;47(9):3766–71. 16936084 10.1167/iovs.06-0153

[10] Prata TS, Palmiero PM, Angelilli A, et al. Iris morphologic changes related to alpha(1)-adrenergic receptor antagonists implications for intraoperative floppy iris syndrome. Ophthalmology. 2009;116(5):877–81. 19410945 10.1016/j.ophtha.2008.12.040 PMC2754294

[11] Leonardi A, Hieble JP, Guarneri L, et al. Pharmacological characterization of the uroselective alpha-1 antagonist rec 15/2739 (SB 216469): role of the alpha-1L adrenoceptor in tissue selectivity, part I. J Pharmacol Exp Ther. 1997;281(3):1272–83. 9190863

[12] Tzamalis A, Matsou A, Dermenoudi M, Brazitikos P, Tsinopoulos I. The role of sex in intraoperative floppy-iris syndrome. J Cataract Refract Surg. 2019;45(1):41–7. 30509747 10.1016/j.jcrs.2018.08.029

[13] Sallam A, Gunasekera V, Kashani S, Toma M. Awareness of IFIS among primary care physicians. J Cataract Refract Surg 2008;34(8): 1230. 10.1016/j.jcrs.2008.01.036 18498979

[14] Taguchi K, Saitoh M, Sato S, Asano M, Michel MC. Effects of tamsulosin metabolites at alpha-1 adrenoceptor subtypes. J Pharmacol Exp Ther. 1997;280(1):1–5. 8996174

[15] Srinivasan S, Radomski S, Chung J, Plazker T, Singer S, Slomovic AR. Intraoperative floppy-iris syndrome during cataract surgery in men using alpha-blockers for benign prostatic hypertrophy. J Cataract Refract Surg. 2007;33(10):1826–7. 17889787 10.1016/j.jcrs.2007.06.033

[16] Chang DF, Osher RH, Wang L, Koch DD. Prospective multicenter evaluation of cataract surgery in patients taking tamsulosin (flomax). Ophthalmology. 2007;114(5):957–64. 17467530 10.1016/j.ophtha.2007.01.011

[17] Takmaz T, Can I. Clinical features, complications, and incidence of intraoperative floppy iris syndrome in patients taking tamsulosin. Eur J Ophthalmol. 2007;17(6):909–13. 18050116 10.1177/112067210701700607

[18] Panagis L, Basile M, Friedman AH, Danias J. Intraoperative floppy iris syndrome: report of a case and histopathologic analysis. Arch Ophthalmol. 2010;128(11):1437–41. 21060046 10.1001/archophthalmol.2010.243

[19] Christou CD, Kourouklidou M, Mataftsi A, Oustoglou E, Ziakas N, Tzamalis A. Silodosin as a predisposing factor of intraoperative floppy iris syndrome (IFIS): an observational propensity score-matching cohort study. Int Ophthalmol. 2022;42(2):393–9. 34609669 10.1007/s10792-021-02054-y

[20] Chang DF, Braga-Mele R. Intraoperative Floppy Iris Syndrome: pathophysiology, prevention and treatment. J Cataract Refractive Surg. 2008;34(11):2008–12. 10.1016/j.jcrs.2008.08.031 19027575

[21] Bucci FA Jr, Fluet AT, Yo C. A randomised prospective masked trial of topical phenylephrine and ketorolac in preventing intraoperative floppy-iris syndrome in patients on tamsulosin undergoing cataract surgery. Clin Ophthalmol. 2012;6:1913–6.

